# Fully covered self-expanding metal stents placed temporarily in the bile duct: safety profile and histologic classification in a porcine model

**DOI:** 10.1186/1471-230X-11-76

**Published:** 2011-06-20

**Authors:** Mihir R Bakhru, Patricia L Foley, Jeremy Gatesman, Timothy Schmitt, Christopher A Moskaluk, Michel Kahaleh

**Affiliations:** 1 Digestive Health, University of Virginia, Charlottesville, VA, USA; 2 Office of Animal Welfare, University of Virginia, Charlottesville, VA, USA; 3 Division of Surgery, University of Virginia, Charlottesville, VA, USA; 4 Pathology, University of Virginia, Charlottesville, VA, USA

## Abstract

**Background:**

Fully covered Self-Expanding metal stents (FCSEMS) have been shown efficacious in palliating malignant biliary obstructions. There is little data analyzing mucosal response to their temporary placement in the bile duct.

**Methods:**

Ten mini pigs underwent endoscopic placement of a FCSEMS (Wallflex, Boston Scientific). FCSEMS were kept in place for three months. At the end of the 3 months, FCSEMS were removed endoscopically. Five pigs were euthanized and their bile ducts harvested. The other five were kept alive for another month post removal. A single pathologist, created a scoring system (to determine degree of inflammation, fibrosis, and epithelial injury), examined all specimens in a blinded fashion.

**Results:**

Four FCSEMS spontaneously migrated in the duodenum. On post mortem examination, mild mucosal thickness was noted in three bile duct specimens while superficial inflammation of the bile duct was noted in five animals. Histologic examination of the bile duct revealed focal acute inflammation in both groups. For the 5 animals euthanized immediately after stent removal, there was a tendency to have superficial mucosal erosion and fibrosis. In contrast, increased chronic inflammation was more commonly seen in the animals 1 month post stent removal, with all animals in this group showing moderate degrees of mononuclear inflammatory cell mucosal infiltrates. No severe inflammatory or fibrotic duct injury was observed in any of the study animals, with degree of injury graded as mild to moderate.

**Conclusion:**

FCSEMS appear to induce minimal tissue overgrowth or fibrosis post placement. Ease of removability and no significant histologic injury are advantages noted with FCSEMS., however, further studies are needed to evaluate treating benign biliary strictures with FCSEMS in humans.

## Background

In the past decade, self-expanding metal stents (SEMS) have been preferred for palliation of malignant obstructive jaundice compared to plastic stents, especially in patients with longer predicted survival [1-5]. They provide prolonged patency, reduced endoscopic sessions, and better drainage due to their larger diameter [6,7]. A major limitation of uncovered SEMS is their occlusion due to tumor growth through the uncovered mesh [8,9]. Partially covered and later fully covered metal stents were created to prevent this occurrence [8]. In terms of patency, recent studies do not demonstrate an advantage for covered metal stents, however, the ability to remove them offer the option of treating benign biliary strictures [10-12].

Partially covered metal stents when studied for benign biliary strictures were associated with migration and epithelial hypertrophy at the level of the uncovered portion of the metal stent [13,14]. Fully covered metal stent with fins had their own set of complications probably related to their high radial force and anti-migratory fins [15-17].

A recently developed FCSEMS with flared ends, without fins might offer a higher resolution rate, if placed temporarily in the bile duct, with limited complications [18]. Currently, there is limited data analyzing mucosal response to their temporary placement in the bile duct.

With the evolution of stents, the pathologic changes that can occur in the bile duct are still not clear. Indeed few animal studies have been done to examine various stents and their effects on the bile duct.

The aim of this study is to establish the safety and histologic effects of temporary FCSEMS placement in a porcine model.

## Methods

The Food and Drug Administration (FDA) and the University of Virginia's Animal Care and Use Committee (ACUC #3756) approved the study. A total of 10 female mini pigs (with acceptable health status and body weight) were acclimated in the vivarium for at least 3 days after arrival.

### Pre-Procedure

The animals were fasted the night before and water withheld the morning before procedures. Animals were pre-medicated intramuscularly with atropine sulphate (0.04 mg/kg) and anesthesia was induced with intramuscular Telazol/Xylazine 4-6/2 mg/kg. Propofol 2-3 mg/kg body weight was administered intravenously to effect to facilitate intubation. Isoflurane titrated to effect was administered to maintain anesthesia. Ventilation was provided via an endotracheal tube.

The animals were placed in recumbence on their left side on a fluoroscopy table. Vitals sign were continuously monitored during the procedure as well as distress or pain.

### Endoscopic Procedure

**Figure 1:**
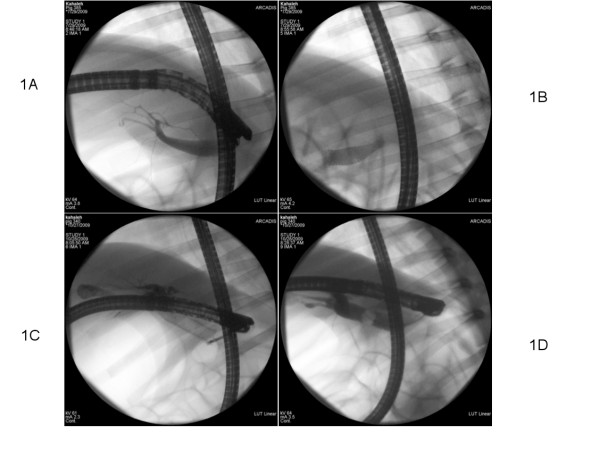
**Ercp Images for Stent Placment and Removal**. **1A**: Endoscopic Retrograde Cholangiography prior to Fcsems Placement **1B**: Fluoroscopy Showing Complete Biliary Decompression after Fcsems Placement **1C**: Rat Tooth Removal of the Fcsems **1D**: Endoscopic Retrograde Cholangiography after Fcsems Removal

A Fuji duodenoscope (ED-250XT5, Fujinon) was used for all endoscopic procedures. Endoscopic Retrograde Cholangiography using standard technique was performed using a triple lumen sphincterotome from Boston Scientific (Ultratome, Natick, MA) by a single endoscopist performing more than 500 ERCPs annually (MK). The bile duct anatomy was delineated under fluoroscopy (Figure 1A), and wire advanced into the biliary tree. The FCSEMS (Diameter of 8 or 10 mm) (Wallflex, Boston Scientific) was deployed under fluoroscopy and endoscopic visualization to confirm good decompression (Figure 1B).

### Post-Procedure Follow-up

After the procedure, the animals were monitored continuously until fully recovered from anesthesia. Yohimbine (0.3 mg/kg) was administered during recovery from anesthesia and ketoprofen (0.5-1.0 mg/kg q 12 hr) with perceived level of need for post-operative analgesia. The animals were monitored daily by a veterinary technician.

Clinical signs including weight loss, pain, or jaundice were monitored as well as daily food intake, respiratory rate, heart rate, and demeanor score (scored as follows: 1 = normal; 2 = slightly to moderately depressed, listless, will stand; 3 = severely depressed, recumbent, will not stand) for the first month following FCEMS placement and weekly thereafter. Subsequently all animals had blood drawn (7 cc) at 2 weeks, 1.5 months, 2.5 months and as needed if the animal showed any clinical signs of malaise. Blood tests included: Total bilirubin (TB), Alkaline Phosphatase (ALK) and Cell blood Count (CBC). The primary investigator (MK) reviewed any abnormal laboratory values.

### Necropsy

At three months, all animals underwent endoscopic FCSEMS removal. The procedure for removal was similar to placement as described above including evaluating FCSEMS position and any tissue reaction or food impaction. The stent was grasped with a snare or forceps under endoscopic control (Figure 1C). Contrast injection via catheter was used to assess ductal anatomy post removal under fluoroscopy (Figure 1D).

**Figure 2:**
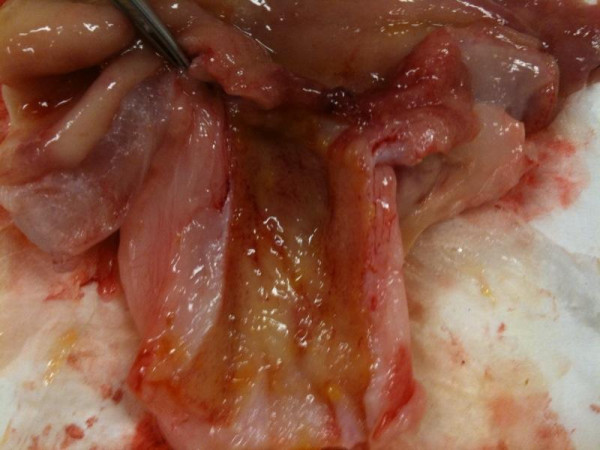
**Macroscopic examination of the bile duct showing superficial inflammation**.

Five of the 10 animals were euthanized (Euthanasia Solution 5 gr/ml 1 cc/10 lbs) and their bile duct harvested for histologic examination. The bile duct was removed from the hepatic bifurcation to the duodenum, including a portion of duodenum and the ampulla. The duct was then opened longitudinally and examined for damage and tissue response (Figure 2). The ducts were then preserved in formalin and sent histological analysis.

The remaining 5 animals were observed for one month. After one month, they underwent repeat ERC before being euthanized and their bile duct harvested for histologic examination.

### Histopathology

**Figure 3:**
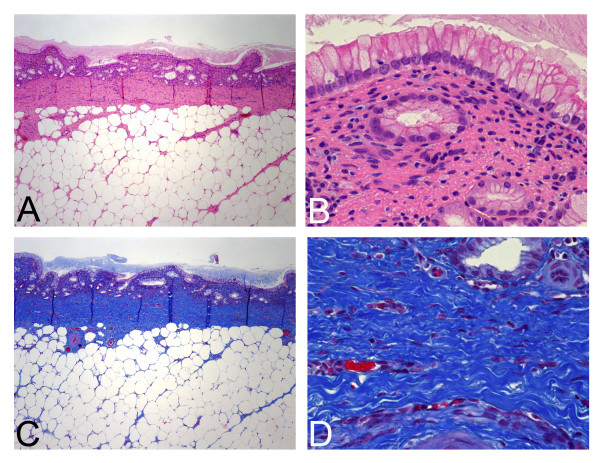
**Normal histology of porcine common bile duct**. **3A**: Intact biliary mucosa overlying a thin fibromuscular wall. Adipose tissue comprises the majority of the adventitia. (original magnification 40×, H&E stain) **3B**: Only scattered mononuclear cells are present in the biliary mucosa. (original magnification 400×, H&E stain) **3C**: Masson trichrome stain highlights normal collagen content (blue staining) (original magnification 40×) **3D**: Normal collagen fibers are thin and reticular in nature. (original magnification 400×, Masson trichrome stain).

The histologic specimens examined were longitudinal sections taken from each bile duct that included the ampulla (Figure 3). The sections were examined with a hematoxylin & eosin (H&E) stain for general histologic features (Figures 3A and 3B). Collagen deposition, as evidenced by increased blue staining on a Masson trichrome stain (Figures 3C and 3D), was interpreted as fibrotic injury.

The dedicated scoring system described in Table 1 was applied by a single pathologist in a blinded fashion to quantify inflammation, fibrosis, and epithelial injury.

## Results

### Initial Placement

All 10 pigs had successful placement of a fully covered WallFlex stent, three pigs received a 10 mm × 40 mm stent and seven received an 8 mm × 60 mm stent. The stents were deployed proximal to the papilla and confirmed in proper position under fluoroscopy. Three of the pigs (Pig #1, 7, 10) had complications during the initial procedure. One pig had a wire perforation on a peripheral branch of the biliary tree noted under contrast injection during stent placement. Two other pigs had a contained perforation of the bulb during a challenging biliary cannulation, requiring the procedure to be postponed for 6 and 8 days, while the animal were treated with omeprazole (20 mg/d) to reduce inflammation. All three pigs were managed conservatively and fully recovered post procedure.

### Clinical Follow-up

For the next three months, monitoring of the pigs included clinical parameters:, i.e.: respiratory rate, heart rate, and demeanor score daily as well as daily food intake for the first month and then weekly. The median change in weight was 16.8 kg with all mini-pigs gaining weight.

Total bilirubin was within normal range for the 2 week, 1.5 month, and 2.5 month interval. All ten pigs had low alkaline phosphatase through each interval with no significant rise. Only one pig had a clinically insignificant decrease in hemoglobin in the first two week interval and then normal at 1.5 and 2.5 months. Eight pigs had low WBC at the two week interval however there was no significant increase in WBC. Pig #1, 2, 4, and 5 were noted to have uneventful stent migration between 41 to 57 days after deployment, all found in the feces of the animal.

### Repeat procedure

**Figure 4:**
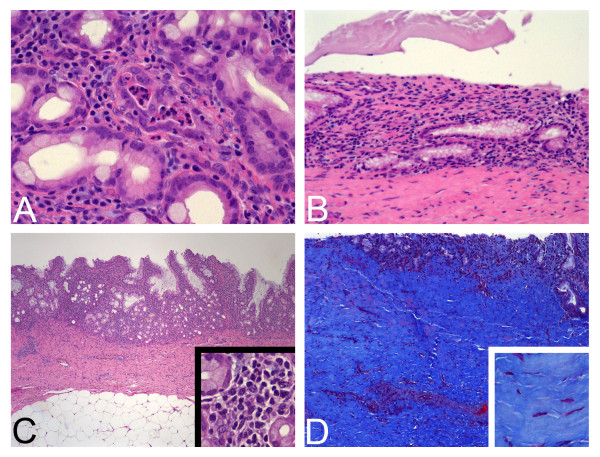
**Examples of acute and chronic injury**. **Figure 4A: Example of acute inflammatory changes**. In the center if the field, polymorphonuclear leukocytes can be seen in the lamina propria and within the lumen of an injured gland. (original magnification 400×, H&E stain) **Figure 4B: Example of epithelial injury**. Typical example of epithelial erosion in where the superficial epithelial layer has been denuded. The deeper glandular mucosa persists and there is no granulation tissue reaction. (original magnification 200×, H&E stain) **Figure 4C: Example of chronic inflammation**. There is an increased population of predominantly mononuclear inflammatory cells, filling the lamina propria of the mucosa (original magnification 40×, inset 400×, H&E stain) **Figure 4D: Example of fibrosis**. Pig #339 showed focal fibrosis that widened the fibromuscular bile duct wall. The main image shows the expansion of the wall by collagen (blue staining). The inset shows thickening and homogenization of the collagen fiber pattern. (original magnification 100×, inset 400×, H&E stain)

At the three-month interval, repeat ERC was performed for stent removal in 6 pigs (4 pigs had stent migration as described above). Five pigs (#3, 6, 7, 8, 9) had stent removal with a rat tooth forceps or snare without difficulty. Pig #10 couldn't have his stent (10 × 40 mm) removed using the rat tooth forceps technique as the stent had migrated proximally into the bile duct. Removal required wire guide balloon dilation with a 10 mm CRE balloon (Boston scientific) allowing the stent to be pulled out from the bile duct. Histological examination revealed 5 (#2, 3, 5, 6, 8) of the 10 animals had focal mild acute inflammation. Focal acute inflammation was seen in both groups of animals (Figure 4A).

For the animals euthanized immediately after stent removal, there was a tendency to have more acute epithelial injury (Figure 4B) in the form of superficial mucosal erosion (4/5 vs 2/5) and mild to moderate mucosal fibrosis (3/5 vs 0/5) (Figure 4C).

In contrast, increased chronic inflammation (Figure 4D) was more commonly seen in the animals 1 month post stent removal (5/5 vs 2/5), with all animals in this group showing moderate degrees of mononuclear inflammatory cell mucosal infiltrates.

The degree of injury was mild to moderate both in acute and chronic inflammation, with no animal showing severe injury. Specifically, there was no abscess formation, deep tissue involvement by chronic inflammation, severe fibrosis, or mucosal ulceration (Table 2).

## Discussion

Fully covered self-expanding metal stents without fins offer adequate biliary drainage in malignant obstruction and potential advantages including reduction in mucosal hyperplasia and migration [12]. These benefits present an attractive option for benign biliary strictures, which can be clinically challenging to treat with serious and often irreversible complications [19,20].

This study analyzes the histologic changes in the bile duct induced by temporary placement of FCSEMS. This particular FCSEMS has a platinol wiring, associated with increased flexibility and radiopacity compared to its predecessor, the partially covered Wallstent [21]. The close cell construction and double coating should reduce tissue ingrowth while the looped and flared ends are intended to reduce tissue trauma and stent migration.

Previous studies have examined the effects of stent placement on the bile duct, but were not designed to provide a histologic grading system for the changes noted. Silvis et al examined surgically placed covered and uncovered wire mesh stents (Wallstent 5 mmx23 mm) in the canine biliary tract and showed mild to moderate cellular infiltration in all animals and marked mucosal hyperplasia with uncovered stents [22]. Van Os et al examined self expanding spiral nitinol stents in a porcine model but had technical limitations in stent placement and removal, related to disparity in stent and duct size or faulty release mechanism (3 of 6 animals). They showed moderate inflammation and fibrosis in 4 of 6 animals euthanized 3 months after stent deployment and mild inflammation in 2 animals kept alive one more month [23]. Further, Ginsberg et al studied bioabsorbable biliary stents in a porcine model, which remained patent up to 6 months with no bile duct integration or proliferative changes, however, stent occlusion and migration was still encountered [24]. More recently, Petersen and colleagues examined the ability to remove partially covered Wallflex stents (within five minutes of deployment) utilizing a porcine model. They demonstrated no significant tissue damage leading to complications [25].

Our objective was to assess the potential changes in the bile duct epithelium after stent placement. Ten mini-pigs were selected and clinically monitored after stent placement for 3 months. Stents were removed or had migrated from all animals at the end of 3 months.

Clinically, there was no evidence of any biliary obstruction in the next three months as evidenced by lack of rising laboratory parameters (bilirubin, alkaline phosphatase, hematocrit, white blood cell count) or clinical findings (jaundice, decreased food intake, pain, change in vital signs or demeanor score). Overall, all pigs gained weight with median change at 16.8 kg from baseline to stent removal.

During the three-month follow up, four of the animals were found with the stent in their feces within two months of placement, without any clinical consequences. The other six had stent removal with no difficulty, except one.

Endoscopic findings at 3 months demonstrated a normal cholangiogram on all animals. Specifically, no biliary injury was noticed on fluoroscopic examination. Gross pathologic examination revealed only mild superficial inflammation in two mini-pigs and mild mucosal thickness in three. The pigs that were euthanized at the 3 month mark included those with stent migration. (Table 1) In addition, of the five mini-pigs kept alive for another month, three had superficial inflammation noted after harvesting the bile duct (Table 2).

The histologic examination confirmed mild acute inflammation in five of the animals, and superficial mucosal erosion (4/5) and mucosal fibrosis (3/5) in the animals sacrificed immediately after stent removal. In contrast, increased chronic inflammation (involving of mucosal and/or submucosal area) was more commonly seen in the animals kept alive after stent removal (5/5). The degree of injury was mild to moderate and no animal had severe injury classified as abscess formation, deep tissue inflammatory involvement, severe fibrosis, or mucosal ulceration.

This study emphasizes the need for a systematic histologic assessment in order to truly understand the implication associated with temporary metal stent placement.

Using a scoring system developed by our pathologist, the bile duct histopathologic alterations after temporary stent placement can be measured and reproduced. This scoring system once validated has the potential to be used for biopsy of the bile duct after stent removal in a patient. Further clinical studies in human subjects using this scoring system could effectively prove the ultimate safety of FCEMS in humans and possibly expand the indication of metal stents to benign biliary strictures.

Limitations to this study include small sample size. Early migration of the stents (noted in 4 pigs #1, 2, 4, 5) was another limitation of this study. All four were analyzed histologically at 3 months. Even though the migration occurred from 41-57 days, respectively, they had similar histologic scores along with the six other pigs. However, because of the early migration, they should be considered as having stent deployed for approximately 1.5-2 months. Importantly, we did not evaluate for post-stenting pancreatitis because of the anatomical difference that would make it less likely for post-ercp pancreatitis in a porcine model. Post-ercp pancreatitis after FCSEMS placement would be a complication to evaluate in human studies, especially in benign biliary strictures.

## Conclusion

Overall, FCSEMS only appear to induce minimal acute inflammation and more importantly minimal fibrosis after stent removal. Chronic inflammation of mild to moderate degree was noted in the animals alive one month longer. The inflammation overall appears to have no clinical effect. Their high rate of migration in the normal bile duct of the pig suggests the necessity to induce a stricture before placement. However, it remains to be determined if FCSEMS can achieve sustained resolution of benign biliary stricture in humans.

## Competing interests

We have received an unrestricted grant from Boston Scientific to perform this study after the Food and Drug Administration approved the study.

Dr Kahaleh has received a research grants from Boston Scientific, Pentax, Emcision, MI tech and Fuji. He is also a consultant for Boston Scientific, Mauna Kea, Axcan Pharma and Xlumina

## Authors' contributions

MB was involved in analyzing the results and writing the manuscript. PF participated in the logistics related to the animal lab. JG participated in the technical care of the animals. TS helped procure the bile duct sample for histologic examination as well as revising the manuscript. CM was the primary pathologist who reviewed histologic specimens, provided the grading scale and scores. MK was the lead person to design and coordinate the study as well as the senior author for the study.

## Pre-publication history

The pre-publication history for this paper can be accessed here:

http://www.biomedcentral.com/1471-230X/11/76/prepub
